# Simple Bone Cyst: A Report of a Rare Case

**DOI:** 10.7759/cureus.116289

**Published:** 2026-09-15

**Authors:** Karthik Rajaram Mohan, Ramachandra Reddy Gowda Venkatesha, Saramma Mathew Fenn, Reethika Rathan, Kumar Appusamy

**Affiliations:** 1 Oral Medicine and Radiology, Vinayaka Mission's Sankarachariyar Dental College, Vinayaka Mission's Research Foundation (DU), Salem, IND

**Keywords:** buccal cortical plate, extended curettage, mandibular molar, posterior mandible, simple bone cyst

## Abstract

A simple bone cyst is an uncommon non-neoplastic intraosseous pseudocyst in the human jawbone. Because it lacks an epithelial lining, we refer to such a cyst as a pseudocyst. For simple bone cysts, several other names exist, such as traumatic bone cyst, solitary bone cyst, progressive bone cavity, hemorrhagic bone cyst, unicameral bone cyst, and extravasation cyst. Usually, routine radiographic examinations discover these cysts, which otherwise are asymptomatic. Unicameral bone cysts tend to gradually increase in size and enlarge along the long axis of the mandibular alveolar bone, causing no cortical expansion or facial deformity. We conducted an accidental case study of a simple bone cyst through routine radiographic examination, which revealed an unusual radiological presentation of cortical plate destruction in the ramus region of the mandible. We also discussed the etiopathogenesis, clinical features, and differential diagnosis. The management of such a simple bone cyst by an extended curettage was discussed as well.

## Introduction

A simple bone cyst is an intraosseous, benign, non-neoplastic cavity that rarely exhibits a straw-colored or serosanguineous discharge [1]. A thin layer of connective tissue lines the cyst inside the bone, but it lacks an epithelial lining [1]. The simple bone cyst's pathophysiology and relationship are debatable and unclear [1]. According to the "trauma-hemorrhage theory," a bone trauma that is not severe enough to fracture results in an intraosseous hematoma. Failure to organize and repair this hematoma may cause it to liquefy and result in a cystic deformity [2]. However, there isn't much evidence in the orthopedic literature to support this theory. It only happens in the diaphysis and metaphysis of long bones in young patients, specifically in the proximal part of the humerus and femur [2]. Other possible causes include interstitial fluid that can't leave the bone because of poor vein drainage, ischemic necrosis of the bone marrow, changes in calcium metabolism, changes in bone metabolism in certain areas, and problems with bone remodeling that lead to osteolysis. Patients with simple bone cysts are often between 10 and 20 years of age [1-4].

Simple bone cyst also affects the maxilla, the mandibular ramus (usually in the premolar and molar areas), and the metaphysis and diaphysis of long bones like the proximal femur and humerus [3,4]. Typically, simple bone cysts remain symptomless and only become visible during radiographs taken for other purposes. In contrast, about 20% of patients experience painless swelling in the afflicted location. When palpating the affected area, if the simple bone cyst develops a secondary infection, one may feel paraesthesia, pain, and soreness when palpating the affected area [3,4]. Patients with simple bone cysts do not have a local odontogenic cause, such as a carious tooth, which can cause intraoral swelling in the gingiva or extraoral swelling resulting in facial asymmetry as a result of the extension and spread of periapical infection into the surrounding fascial planes. Intraoral clinical examination of patients with simple bone cysts does not reveal a carious tooth or a fine enamel crack, which is invisible to the naked eye during a clinical examination. Patients with simple bone cysts usually give a history of an episode of trauma [3,4].

## Case presentation

A 19-year-old female reported to the outpatient department with a chief complaint of mild intermittent pain and discomfort in her lower right back tooth region for the past month only during the mastication of foods. On eliciting the history of presenting illness, the patient gives a history of trauma to her lower right back tooth one month ago following an accidental biting on a small stone while taking her meals. Her medical history revealed no significant findings. Her vitals were within biological ranges. The patient was afebrile. An extraoral examination revealed no facial asymmetry. A clinical assessment of her neck revealed no submandibular lymphadenopathy or other regional lymph nodes. An intraoral examination of her right mandibular posterior tooth did not reveal any visible fracture or discoloration of the right mandibular posterior teeth (Figure 1).

**Figure 1:**
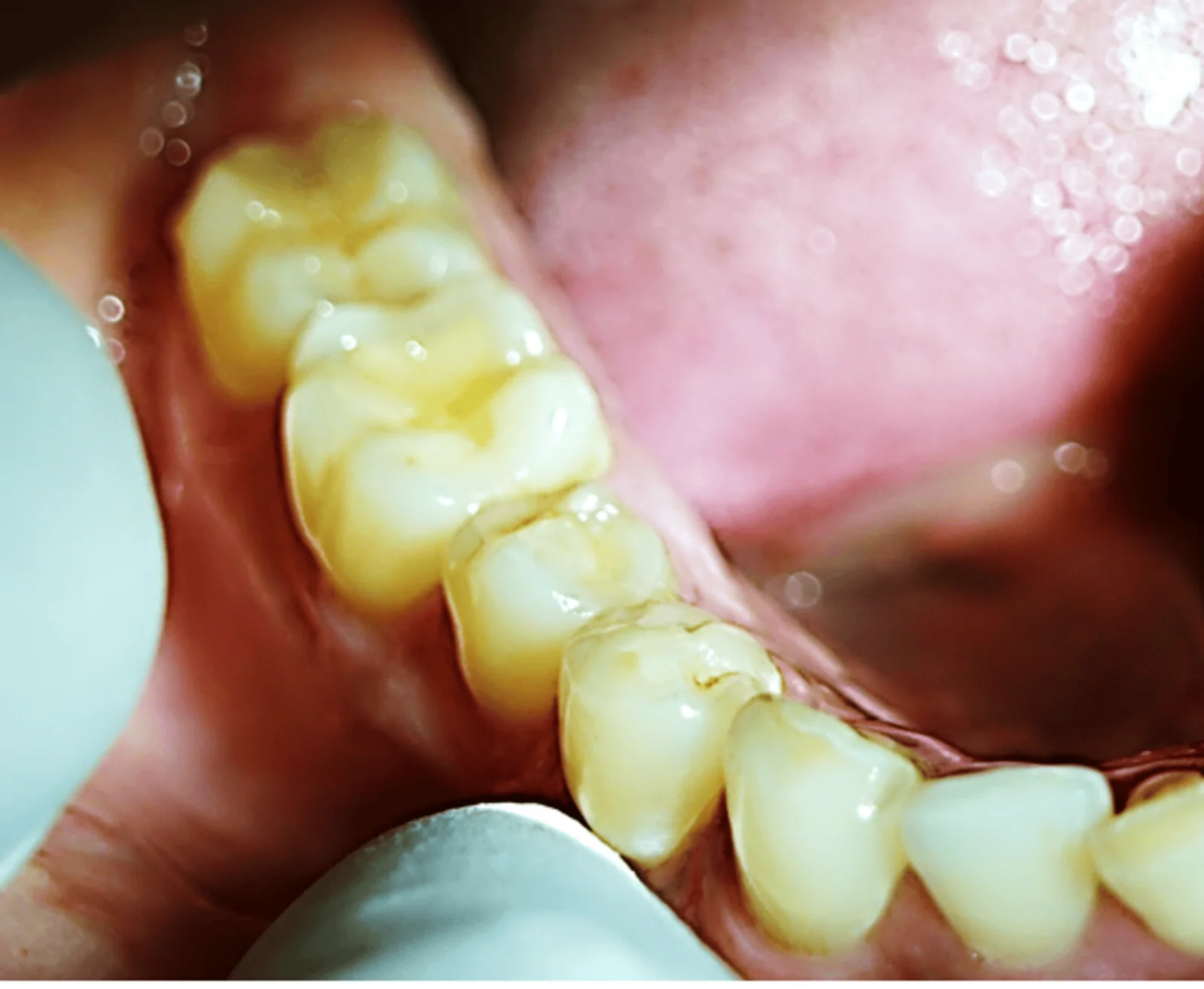
Intraoral clinical examination revealed no carious cavity or visible swelling in relation to the right mandibular premolars and first and second molar teeth

We performed the rubber wheel test on her right mandibular posterior teeth to identify fine enamel cracks that might not be immediately visible to the naked eye. However, she revealed no discomfort or pain in her right mandibular posterior teeth. On an intraoral periapical radiograph of her right mandibular second molar, we saw a scalloping radiolucent border near the top of the mandibular alveolar bone, which was near the back of the right mandibular second molar tooth (Figure 2).

**Figure 2:**
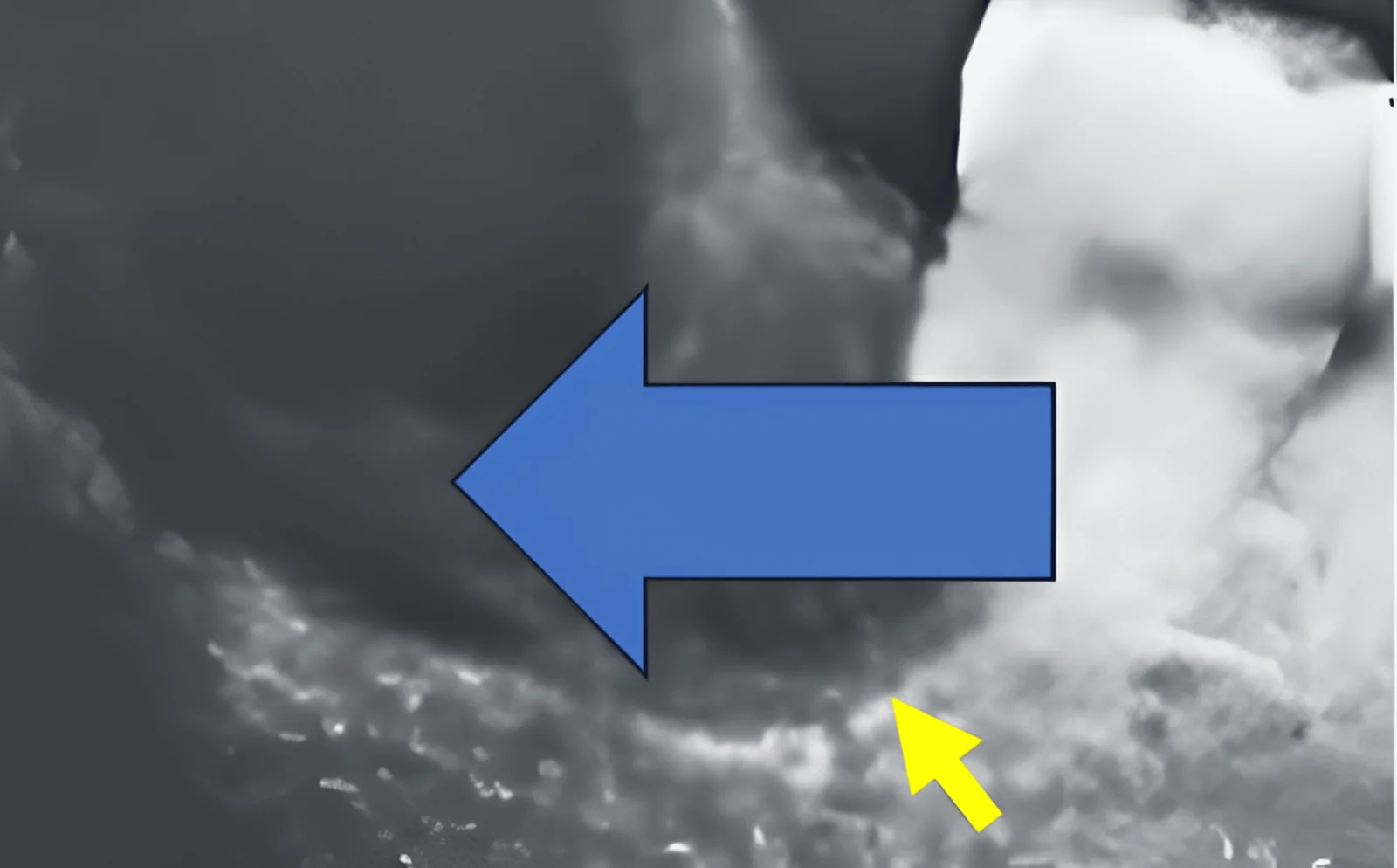
Intraoral periapical radiograph revealed a well-defined radiolucent area in relation to the right mandibular second molar region (blue arrow) bordered by a scalloping border between the teeth roots (yellow arrow)

The orthopantomography image revealed a sizeable radiolucent lesion measuring 1.5x2 cm extending posteriorly from the apex of the root of the mandibular first molar region to the right-side ramus of the mandible with scalloping near the superior border of the mandibular alveolar bone distal to the right mandibular second molar region with no intervening internal septa (Figure 3).

**Figure 3:**
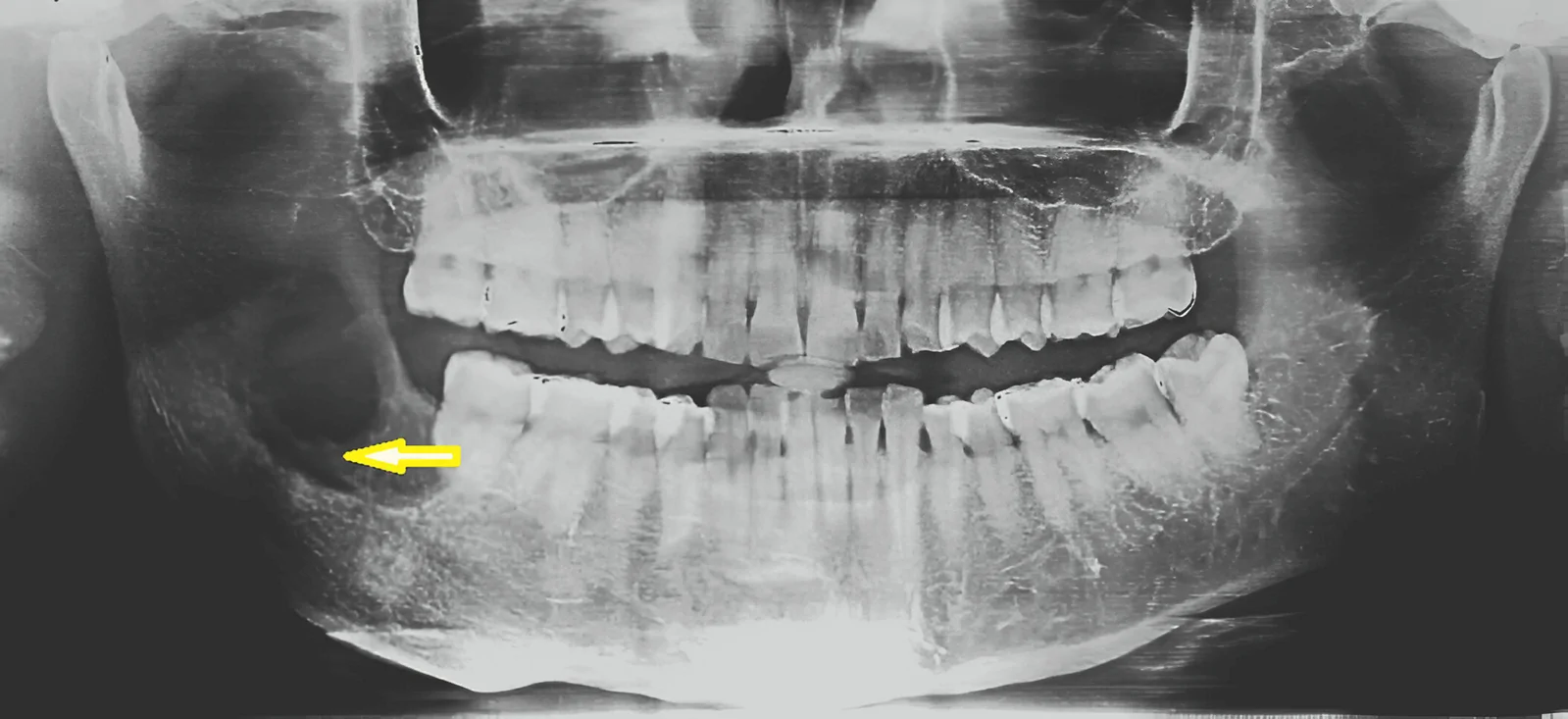
OPG revealed a scalloping border extending from the distal surface of the root of the mandibular second molar (yellow arrow) OPG: orthopantomograph

An axial section of the computed tomography scan image shows a centrally located hypointense area within the buccal and lingual cortical bone destruction with thinning of the cortex (Figure 4).

**Figure 4:**
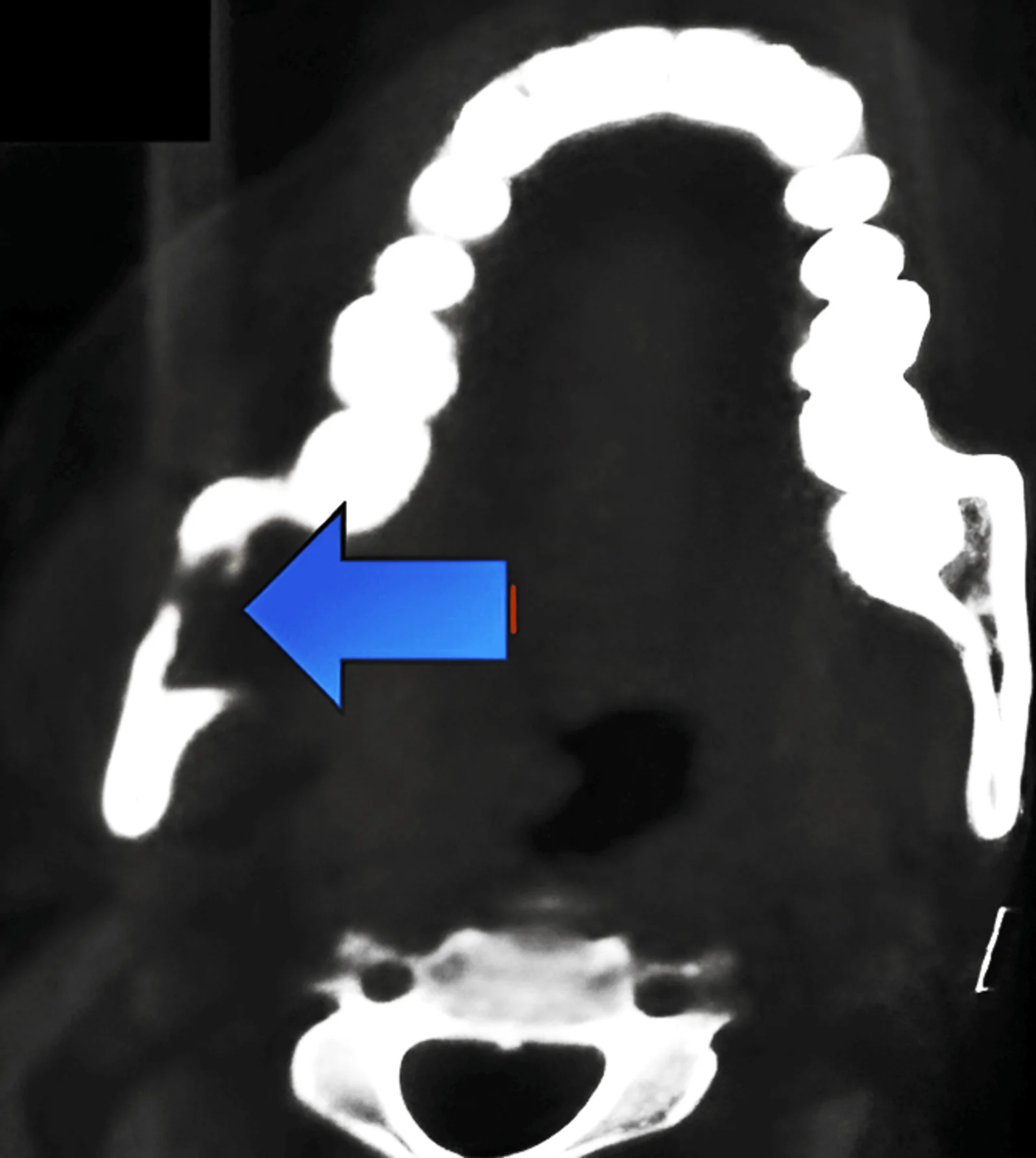
Axial section CT scan revealed a hypodense area in relation to the ramus region of the mandible (blue arrow) CT: computed tomography

The three-dimensional reconstructed image of the CT scan revealed buccal and lingual cortical plate destruction above the level of the right submandibular fossa in the region of the right-side ramus of the mandible (Figure 5A, 5B).

**Figure 5:**
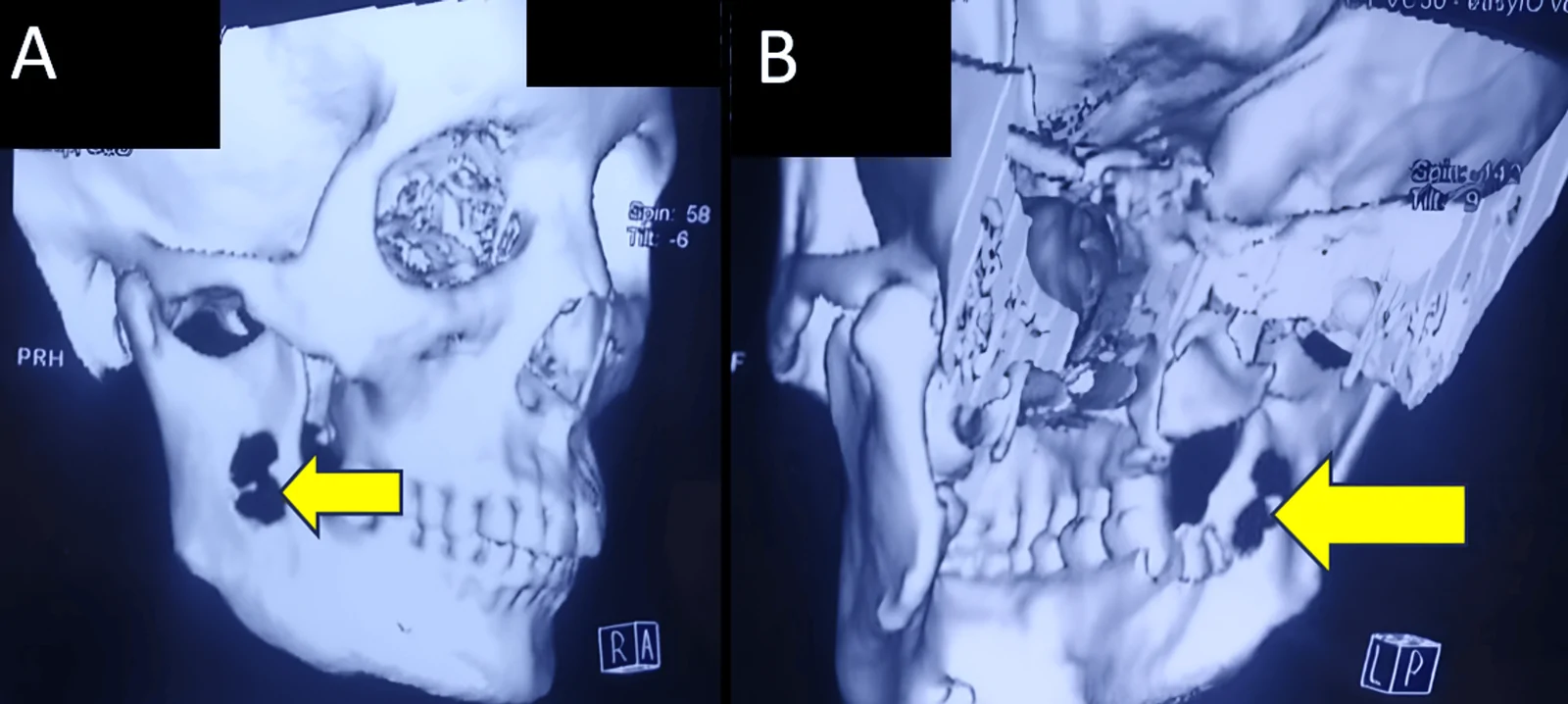
3D reconstructed CT images revealed (A) buccal cortical destruction and (B) lingual cortical destruction in the ramus region in relation to the right mandibular second molar region (yellow arrow) 3D: three-dimensional; CT: computed tomography

The right mandibular posterior teeth responded to thermal pulp tests using heated gutta-percha sticks and electric pulp tests. Aspiration done using a disposable syringe with a 22-gauge needle revealed empty contents with no blood, straw-colored, dirty white cheesy, or serosanguineous fluid. The differential diagnosis of simple bone cysts includes Stafne bone defect, radicular cyst, odontogenic keratocyst, central giant cell granuloma, aneurysmal bone cyst, and ameloblastoma. The radiographic differential diagnosis for simple bone cysts includes Stafne bone defect, odontogenic keratocyst, and unicystic ameloblastoma. Stafne bone defects usually arise below the level of the inferior alveolar canal region in the submandibular fossa region. In contrast, a simple bone cyst occurs above the level of the submandibular fossa region, above the inferior alveolar canal region. The affected tooth is usually non-vital in a radicular cyst, whereas the tooth near a simple bone cyst is generally vital. Odontogenic keratocysts tend to grow along the axis of the bone with very little expansion but resorb and displace the teeth. On aspiration, the aneurysmal bone cyst reveals a blood-soaked or filled sponge.

While simple bone cysts do not have proper internal bony septa, they can appear on radiography as internal bony septa due to prominent scalloping of the endosteal surface of either the buccal or lingual plates. The central giant cell granuloma exhibits evidence of a very fine granular pattern or a wispy, ill-defined internal bony septum. From a lateral view of the jaw, the scalloping creates bone ridges that resemble septa. The pathological migration of teeth and the enlargement of the buccolingual cortical plate, along with resorption on the external surface of the teeth, are caused by ameloblastoma. Still, simple bone cysts do not result in these conditions. We performed an inferior alveolar nerve block under 2% lidocaine local anesthesia. We surgically explored the area distal to the mandibular second molar by elevating the mucoperiosteal flap using a periosteal elevator. During surgery, the top edge of the mandibular alveolar crestal bone region farthest from the right mandibular second molar was examined. It showed an empty space with no epithelial lining or strange, cheesy, or straw-colored discharge (Figure 6).

**Figure 6:**
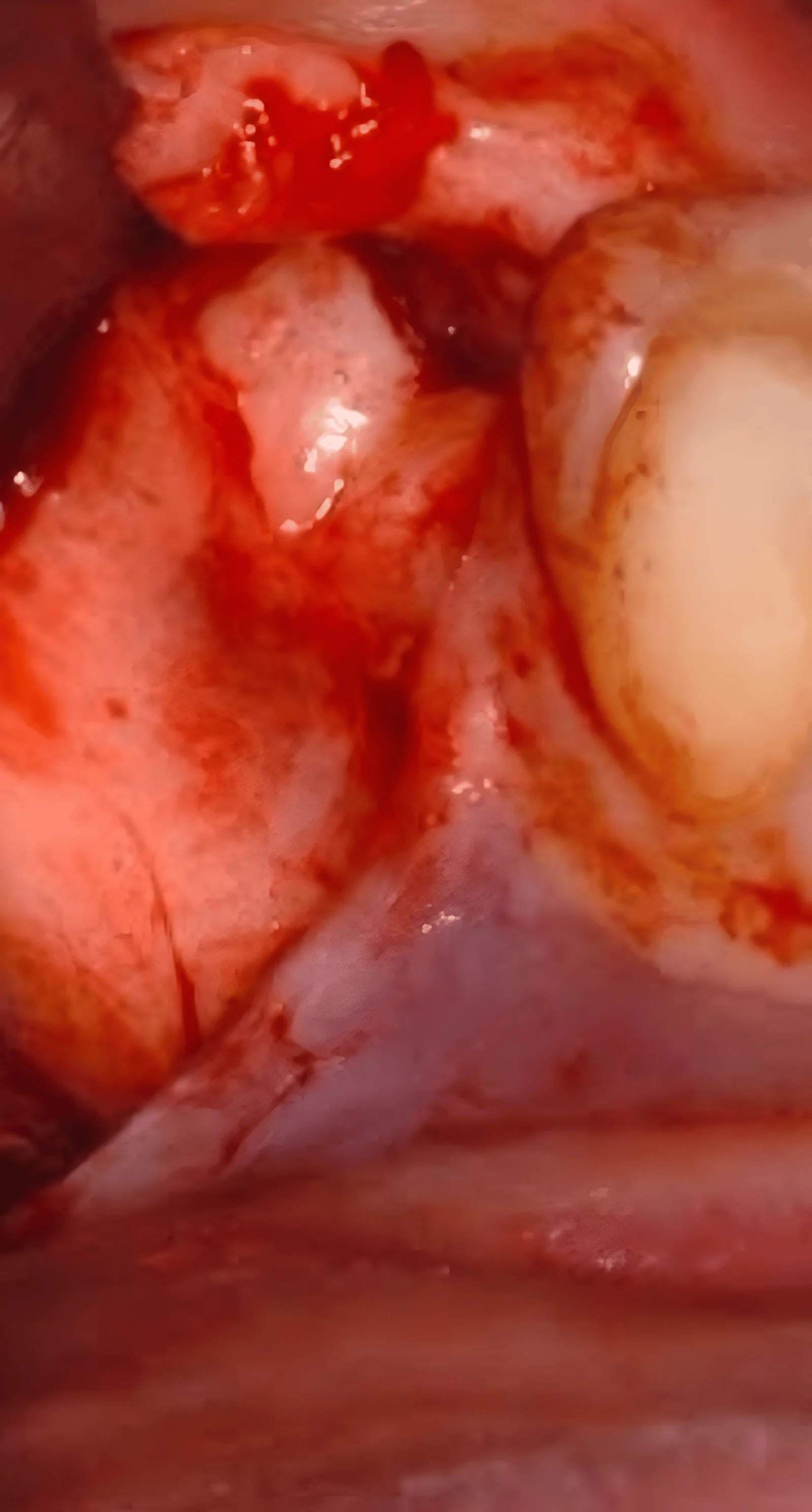
Surgical exploration of the site revealed an empty bone cavity

The above clinical and radiographic findings lead to the final diagnosis of a simple bone cyst. We irrigated the surgically explored area with normal saline (NS) (0.9% sodium chloride), used a Lucas curette with 2.5 mm ends to extend the periapical curettage and induced bleeding, and then used an absorbable Vicryl suture to close up the mucoperiosteal flap. After six months, we conducted a follow-up evaluation using postoperative orthopantomography, which revealed the affected area had resolved (Figure 7).

**Figure 7:**
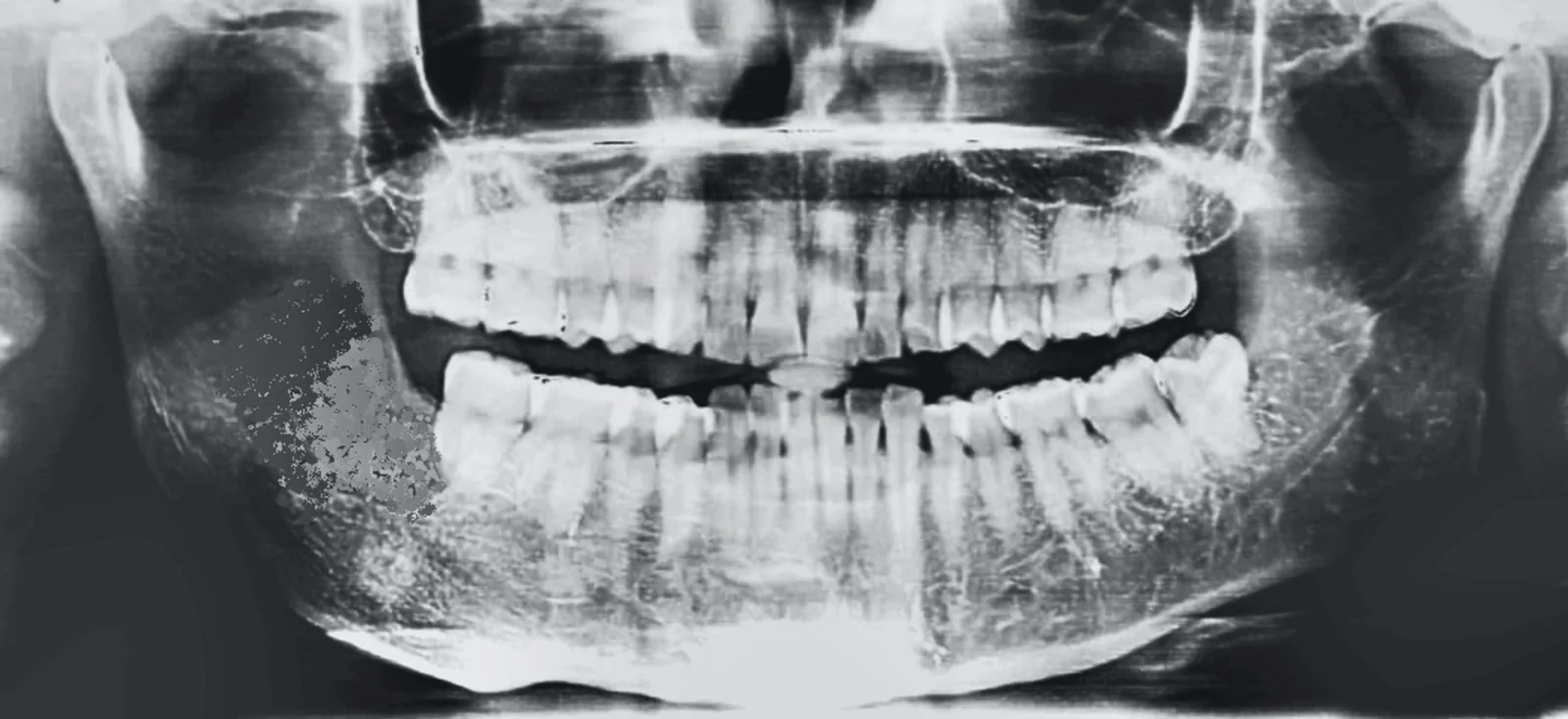
Follow-up OPG after six months revealed the resolution of the affected area in the right mandibular second molar region OPG: orthopantomograph

A simple bone cyst has an excellent prognosis. If left untreated, it does not cause pathological jaw fractures, unlike radicular cysts. Our case showed a good prognosis after extended curettage and radiographic follow-up evaluation after six months. No recurrence of the lesion was reported.

## Discussion

In a significant milestone and groundbreaking moment, Lucas unveiled the first simple bone cyst in 1929 [1]. Rushton explained that this important discovery helped us understand a pseudocyst that doesn't have an epithelial coating, doesn't show signs of acute or chronic infection, and can be full of fluid or empty [1,2]. In a radiographic examination, Aoki et al. further explained that simple bone cysts imitate Stafne bone defects, which are steepened submandibular fossas that mimic a cyst-like radiolucency [3].

Therapy for a simple bone cyst is not necessary unless there is an imminent fracture; the bone cyst is enormous, and it causes symptoms or deformities [4]. It's worth noting that simple bone cysts are known by various other names, such as solitary bone cysts, unicameral bone cysts, traumatic bone cysts, hemorrhagic bone cysts, progressive bone cavities, idiopathic bone cysts, and extravasation cysts [5]. Researchers postulated several etiological hypotheses for their occurrence [5-7].

The etiology of simple bone cysts is a subject of ongoing research and debate. Cohen proposed the idea that the cyst forms because interstitial fluid can't leave the bone because there isn't enough venous drainage because the venous sinusoids aren't draining lymphatic fluid. Therefore, this barrier seems to stop interstitial fluid from leaving the bone and getting trapped. This builds up pressure and activates osteoclasts, which break down the bony trabeculae and create cysts [6]. A developmental anomaly that incorporates synovial tissue intraosseously leads to the development of simple bone cysts, which are synovial cysts [7]. These intricate and diverse hypotheses beckon us to delve deeper into understanding simple bone cysts.

Higher intramedullary osseous pressures cause ischemic blood flow within the bone, according to Chigira et al. [8]. This supports the common theory that these simple bone cysts are caused by blocked venous drainage in the bone [8].

Other reasons could include "local ischemia," "intramedullary bleeding," "local imbalance in bone growth," "a change in calcium metabolism," "a change in bone metabolism resulting in osteolysis," or a mix of these [8]. These hypotheses offer intriguing insights into the potential causes of simple bone cysts, sparking further research and discussion in the field. Vayego-Lourenco suggested a TP53 mutation for the appearance of unicameral bone cysts [9].

We think that biting down on a stone by accident may have caused ischemic blood flow in the alveolar bone near the second and third molars in the lower jaw. This could have led to the formation of a simple bone cyst in that area. A sudden episode of trauma from accidental biting on a hard stone during meals must have caused the formation of a simple bone cyst. But our case was contradictory to the study by Flores et al., who reported 42 cases of solitary bone cysts without any history of trauma [10]. 

Most experts agree that a simple bone cyst can be caused by a problem with the blood vessels that leads to localized post-hemorrhagic ischemia and osseous aseptic necrosis [11].

Routine radiographic examinations often reveal inadvertent simple bone cysts. Such cysts are intraosseous pseudocysts [12]. The definite diagnosis of such a surgery invariably finds an empty bone cavity, as in our case, confirming a simple bone cyst. Less than 10% occur in the maxilla or mandible gnathic bones, with the posterior ramus involving the molar-premolar region being the most common location. Simple bone cysts also occur in the metaphysis or diaphysis of long bones, such as the proximal femur or humerus. Routine dental radiographs typically detect simple bone cysts, which are typically asymptomatic. A clinically simple bone cyst progresses along the long axis of the affected alveolar bone and hence does not cause much cortical plate expansion. A clinically simple bone cyst does not cause significant cortical destruction. In our case, the axial and three-dimensional reconstructed CT scan image clearly displays cortical destruction. Radiographically, simple bone cysts are often polymorphic, variably sized radiolucent lesions with smooth, well-defined, and poorly ill-defined borders. A simple bone cyst is identified on X-rays by a "scalloped superior cortical margin extending between the roots of the teeth."

This is because the lesion usually scallops between the roots of the teeth, and root resorption is not common, as was seen in our case. "The lesion usually leaves the lamina dura of the affected teeth intact or only partially disrupted." There is no displacement of an inferior alveolar nerve canal or pathological migration of the affected tooth. 

The lamina dura of the teeth remained intact in our case, and there was no evidence of pathological migration of adjacent teeth or displacement of the inferior alveolar canal.

The curvature of the bone marrow's intervening septa, along with a radiolucency and a scalloping radiopaque border distal to the right mandibular second molar, further supported the diagnosis of a simple bone cyst in our case.

Jordanov described the "rising bubble sign" as a new radiological feature that aids in diagnosing simple bone cysts [12]. Struhl et al. described the "fallen fragment sign" as a radiological sign resulting from a pathologic fracture of the cortical bone fragments, which may dislodge and gravitate towards the dependent portion of the simple bone cyst [13]. When a solitary bone cyst affects the mandibular ramus, the patient usually gives a history of recurrent lower lip hypoaesthesia. The radiological feature often shows cortical perforation [14]. Occasionally, such a simple bone cyst may extend into the ramus, as reported by Hosseini and Strabbing et al. in our case [13,14]. Altermatt et al. suggested using tricalcium phosphate ceramic to treat simple bone cysts [15]. According to Lokiec et al., percutaneous autologous bone marrow grafts are used to treat simple bone cysts [16]. Hou et al. stated that tissue-engineered bone grafts help heal solitary bone cysts and have better safety and efficacy than allogeneic bone grafts [17]. Scaglietti et al. first advised using methylprednisolone acetate injections to treat simple bone cysts [18]. Many simple bone cysts persist years after their exploration and diagnosis, according to Resnick et al. Unresolved lesions did not get larger or become harmful [18]. Scaglietti et al. said that a simple bone cyst that formed during the growth spurt would heal more or less quickly if control X-rays were taken every two to three months and corticosteroid (methylprednisolone acetate) injections were given over and over to areas where bone regeneration was slow in places where there were still holes in the bone [19]. Researchers have proposed allogenic materials like gel foam, lyophilized bone, or hydroxyapatite as fillers for the bone cavity when osteo-integrated implant rehabilitation is required. Simple curettage of the bone cavity walls likewise yielded positive results, as evidenced by progressive osseous regeneration within six months and no sign of recurrence two years following the procedure [20].

Clinical significance

One cannot overstate the clinical significance of simple bone cysts. These cysts, which are asymptomatic, typically occur occasionally during routine radiography examinations. However, when they do cause symptoms, the impact can be significant. The frequency is higher in females with a mean age of 42 and in the mandible, frequently in the ramus and posterior jaw. Most of the time, exploring a surgical lesion reveals an empty cavity, and the patient typically has some history of trauma. Surgical exploration and careful curettage help fill the empty cavity with blood, resulting in the organization of blood clots and the healing of the affected area. This underscores the importance of early detection and appropriate treatment in managing simple bone cysts.

Spontaneous resolution of simple bone cysts occurs following a diagnosis based on epidemiological, clinical, and radiographic characteristics. The only drawback is that choosing this protocol requires considering the length of time needed for the lesion to fully resolve. The histopathologic features are not typical; hence, radiographic and surgical observations are the primary sources of information for the diagnosis. On rare occasions, simple bone cysts heal on their own. Because new, immature bone is formed, a biopsy and study of a healing simple bone cyst may mistakenly reveal the existence of an ossifying fibroma or fibrous dysplasia. Regular clinical and radiological follow-up is necessary to ensure successful treatment of such a simple bone cyst.

## Conclusions

A radiographic evaluation guides the diagnosis of a simple bone cyst. The simple bone cyst has a characteristic scalloping border between the roots of the affected teeth. Simple bone cysts can be asymptomatic or cause mild discomfort when infected. Extended curettage or corticosteroid injections aid in osseous regeneration and promote healing in the treatment of a simple bone cyst. Simple bone cysts can be treated with extended curettage, a local injection of corticosteroid, or a bone graft to speed up healing, repair, and fill the hole in the bone that the cyst left behind. Unlike other cysts, these cysts don't need invasive surgeries like segmental or en bloc resection. A follow-up radiological evaluation of simple bone cysts is required to check for osseous regeneration.
